# Principles and Illustrations of Morbid Anatomy; Adapted to the Elements of M. Andral, and to the Cyclopædia of Practical Medicine, &c.

**Published:** 1834-10-01

**Authors:** 


*Principles and Illustrations of Morbid Anatomy; adapted to the Elements of* M. Andral, *and to the Cyclopædia of Practical Medicine, &c.* By J. Hope, m.d., f.r.s., Physician to the St. Marylebone Infirmary, &c.—London, 1834. 8vo. Parts XI. and XII. Pp. 57 and xi. Coloured plates.

Many intelligent practitioners are of opinion, that morbid anatomy has for some years been cultivated with a zeal quite disproportioned to its practical value. They remark, that post-mortem examinations frequently lead to conclusions as to the treatment of disease which experience proves to be false, but more frequently lead to no conclusion at all; and that the benefit to be derived from these investigations will be reaped (if at all) by a distant posterity; for that, in our time, medicine must remain as it is,—not a logical system, but a refined empiricism. We do not, however, quite agree with these objectors: for, in the first place, we do not see why men of science should not accumulate facts to be profitably employed by their grandchildren, just as the provident husbandman plants oaks as well as cabbages; and moreover, it is to be hoped, if not expected, that the indefatigable industry of our dissectors will ultimately be rewarded with discoveries immediately applicable to the art of healing. It must be admitted, in addition, that great bodies of men, as well as individuals, are sometimes urged on by a spirit of enthusiasm, and pursue some favourite branches of knowledge with an exclusive zeal, which would be pernicious, were it constant; but which, lasting only for a few years, produces results which could never arise from the toils of more cold and calculating students. It is to be desired, however, that every practitioner who devotes himself for a series of years to the minute examination of dead bodies, should afterwards apply the knowledge which he has thus gained to the advantage of living ones. This duty is especially incumbent on those who, like the industrious author before us, have the charge of a hospital or infirmary: it is theirs to show their less fortunate brethren what are the results of the treatment of disease on a large scale, and to dissipate their errors by the irresistible evidence of myriads of successful cases. We trust, therefore, now that Dr. Hope is released from the heavy but useful task which he had assigned himself, he will devote himself to the execution of a practical work, for which the honourable post which he occupies will afford him abundant materials.

The eleventh Number of the Principles and Illustrations of Morbid Anatomy contains the remainder of the diseases of the Uterus, and the diseases of the Kidney, Bladder, and Spleen; of which, the last three are despatched with great brevity. The twelfth and last Part gives an account of the diseases of the Brain and Spinal Cord. We must content ourselves with a single extract.

" *Cerebritis, or Inflammation of the Substance of the Brain.* Considerable difficulty is experienced in ascertaining the existence of inflammation of the substance of the brain in its early stages, in consequence of the natural standard of vascularity being different at different ages, and in consequence of certain diseases, certain modes of death, and several other circumstances, giving rise to congestion, which may be mistaken for inflammation. It will therefore be desirable to premise the present subject with a brief sketch of the natural appearances of the brain, and of the aspects and causes of congestion.

" *Natural Appearance of the Brain.* In adults, the colour of the cortical substance of the cerebral hemispheres is aptly compared to weak coffee, mixed with much milk, as in Fig. 241, from a female æt. nineteen. In infancy and early youth, the vascularity being greater, the colour is rather more purple, and resembles chocolate with milk. In elderly people, a diminution of vascularity, by withdrawing the pink intermixture, leaves the substance greyer and paler; and, in very advanced age, it acquires a slight yellowish tint, which, however, sometimes appears prematurely at a much earlier period.* When the pia mater is torn off, the surface of the grey substance is speckled over with red dots, arising from the rupture of the meningo-cephalic vessels, the dots being more numerous in proportion as the brain is more vascular: the interior of the grey substance also presents a few dots and streaks, which are less abundant, or at least less apparent, than those of the white substance.

"The grey substance is composed of three distinct layers: the first, or external, of a whitish grey; the second, which is very thin, of a dirty white; and the third, which is the thickest, of a more leaden grey. Dr. Bright states that six layers may occasionally be discerned. The layers are marked by natural or accidental differences of colour.

"The white substance of the cerebral hemispheres is usually called milk-white in young people and adults; but, on repeatedly comparing it with white paper, I have found it to present a delicate pink tinge, which is deeper in proportion as the subject is younger, and also a faint yellow tinge, very much as in Fig. 241, produced by a very faint carmine wash, and, over it, an ochre wash. After the age of fifty the pinkness vanishes, leaving the white more pure than at any other period of life; in old age a yellower tint super-

* "Andral, Path. Anat., vol. ii., p. 712; and Cazauvieill, Recherches Anatomico-Physiologiques sur l'Encéphale, considéré chez l'Adolescent, l'Adulte, et le Vieillard."

venes. On making a section, a few scattered dots of blood ooze out of the cut vessels, and are intermixed with a few short capilliform streaks, as if from vessels cut longitudinally. The blood from a few of the larger vessels diffuses itself, and forms small blotches. The number of these red specks, streaks, and blotches is greater in children than in adults, and in these than in the aged.

" *Congestion.* Acute disease, attended with increased arterial action, augments the injection of the brain, while chronic disease, producing anæmia, diminishes it. Diseases attended with obstruction to the return of the blood from the head, as asthma, organic affections of the heart, &c. augment cerebral congestion; and, on the same principle, it is augmented by death from asphyxia or convulsions. It is likewise increased by hypertrophy of the left ventricle, which causes a preternatural determination to the brain. After death, if the head be placed in a dependent position, very considerable injection and even uniform redness may be occasioned by the gravitation of the blood, and its transudation through the coats of the vessels. The same transudation may occur if the examination be delayed till putrefaction commences, especially when the weather is hot. Cerebral congestion is diminished by opening the chest before the head, and thus allowing the blood to drain off by the jugular veins. The French mode of opening the cranium by the hammer causes redness and ecchymosis along the line of the fracture. It is scarcely necessary to add the familiar fact, that, when the surface of the brain, divested of its membranes, is exposed for some time to air, its redness is greatly increased in consequence of the venous blood becoming oxygenized; and the same change is sustained, though in a less degree, by the interior when sliced and similarly exposed." (P. 282-284.)

The extreme beauty of the plates is kept up to the last; and we cannot part from this work, so creditable to the talents, as well as the industry of Dr. Hope, without again cordially recommending it to our readers.

